# Minimally Invasive Trans-Axillary versus Full Sternotomy Mitral Valve Repair: A Propensity Score-Matched Analysis on Mid-Term Outcomes

**DOI:** 10.3390/medicina60010029

**Published:** 2023-12-23

**Authors:** Olimpia Bifulco, Pietro Giorgio Malvindi, Paolo Berretta, Leonardo Brugiatelli, Mariano Cefarelli, Jacopo Alfonsi, Alessandro D’Alfonso, Carlo Zingaro, Marco Di Eusanio

**Affiliations:** Cardiac Surgery Unit, Lancisi Cardiovascular Center, Ospedali Riuniti delle Marche, Polytechnic University of Marche, 60126 Ancona, Italym.dieusanio@univpm.it (M.D.E.)

**Keywords:** mitral valve, mitral valve repair, minimally invasive mitral valve surgery

## Abstract

*Background and Objectives*: Minimally invasive cardiac surgery is an established approach for the treatment of heart valve pathologies and is associated with excellent technical and early postoperative outcomes. Data from medium- and long-term longitudinal evaluation of patients who underwent mitral valve repair (MVr) through transaxillary approach (TAxA) are still lacking. The aim of this study is to investigate mid-term results in patients who underwent TAxA MVr. *Materials and Methods*: Prospectively collected data of patients who underwent first-time MVr for MV regurgitation between 2017 and 2022, were reviewed. A total of 308 patients received TAxA, while in 220 cases, traditional full sternotomy (FS) was performed. Concomitant aortic and coronary artery bypass grafting (CABG) procedures, infective endocarditis or urgent operations were excluded. A propensity match (PS) analysis was used to overcome preoperative differences between the populations. Follow-up data were retrieved from outpatients’ clinic, telephone calls and municipal administration records. *Results*: After PS-matching, two well-balanced cohorts of 171 patients were analysed. The overall 30-day mortality rate was 0.6% in both cohorts. No statistical difference in postoperative complications was reported. TAxA cohort experienced earlier postoperative extubation (*p* < 0.001) with a higher rate of extubation performed in the operating theatre (*p* < 0.001), shorter intensive care unit (ICU) stay (*p* < 0.001), and reduced hospitalization with 51% of patients discharged home (*p* < 0.001). Estimated survival at 5 years was 98.8% in TAxA vs. 93.6% in FS cohort (Log rank *p* = 0.15). The cumulative incidence of reoperation was 2.6% and 4.4% at 5 years, respectively, in TAxA and FS cohorts (Gray test *p* = 0.49). *Conclusions*: TAxA approach for MVr was associated with low rates of in-hospital mortality and major postoperative complications being furthermore associated with shorter mechanical ventilation time, shorter ICU stay and reduced hospitalization with a higher rate of patients able to be discharged home. At mid-term, TAxA was associated with excellent survival and low rate of MV reoperation.

## 1. Introduction

At specialized centres, minimally invasive cardiac surgery has become the preferred approach over conventional full sternotomy (FS) for the treatment of mitral valve pathology meeting the growing demand for a quicker recovery and excellent technical results [1,2]. Alongside a reduced hospitalization, further benefits of this approach include decreased postoperative pain, improved cosmesis and lower incidence of deep wound infection. However, while several studies have reported the safety and efficacy of minimally invasive mitral valve surgery, the adoption of this approach is slowly spreading worldwide, due to several concerns including the steep learning curve, the potential high costs related to specialized equipment, and the lack of mid- and long-term surgical evidence [3,4,5]. Among several minimally invasive approaches using right mini thoracotomy, we favour the trans-axillary access (TAxA) and a simplified conduct with direct view of the valve and subvalvular apparatus with no need for video assistance or other specialised tools [6]. We have previously reported the early results of our experience with TAxA mitral valve surgery [7]. The aim of this study was to focus on mid-term results in patients who underwent TAxA mitral valve repair.

## 2. Materials and Methods

### 2.1. Study Design and Ethical Approval

This is a single-centre retrospective study on prospectively collected data. All data including patient’s baseline characteristics, intraoperative, and postoperative features were retrieved from the internal database of Cardiac Surgery Unit at Lancisi Cardiovascular Centre—Polytechnic University of Marche—Ancona (Italy) (approval from Comitato Etico Regionale Marche 2019 361).

### 2.2. Population

All consecutive patients who underwent first-time mitral valve repair for mitral valve regurgitation between January 2017 and June 2022, were included.

Concomitant tricuspid valve repair and/or rhythm surgery (pulmonary vein isolation/MAZE procedure) did not represent an exclusion criterion [8], while patients who underwent concomitant aortic and CABG procedures, and operated on for infective endocarditis or on urgent/emergency basis were not included.

Mitral valve procedures were performed using either the trans-axillary or classic full sternotomy approach. Propensity match analysis on baseline characteristics was provided to create two well-balanced patient cohorts. The patients were followed by outpatients’ clinic and telephone calls.

Mortality data were derived from the municipal administration records and mitral valve reintervention events were retrieved by cross-referencing the registry’s database.

### 2.3. Definitions

Preoperative characteristics were reported according to EuroSCORE definitions [9]. Post-operative complications and 30-days mortality were recorded referring to VARC-2 criteria [10]. Hospital stay was defined by the postoperative days spent at our Cardiac Surgery department (day 0 is the day of surgery). Mid-term outcomes were defined as survival and reintervention on mitral valve at 5 years.

### 2.4. Echocardiographic Assessment

Before all surgical procedures, the assessment of mitral valve pathologies and the grading of valve regurgitation were evaluated by transthoracic (TTE) or trans-oesophageal (TOE) echocardiogram in our echocardiography core laboratory. TOE was performed intraoperatively in all the patients as a TTE evaluation before hospital discharge.

### 2.5. Surgical Techniques

According to mitral valve disease, different repair techniques, such as leaflets resection, implantation of artificial chords and annuloplasty rings were used either via trans-axillary or via full sternotomy.

#### 2.5.1. Trans-Axillary Access

As previously described [11,12], under general anaesthesia, right jugular vein was cannulated before surgical draping. The cardiopulmonary bypass was conducted maintaining normothermia after cannulation of the common femoral artery and the femoral vein, using the Seldinger technique and TOE guidance after surgical cut-down.

With the patient in supine position, a 4 to 5 cm skin incision was made in the right anterior axillary line at the level of the 4th intercostal space. A soft tissue retractor was used to limit rib spreading. The aorta was occluded using a flexible clamp introduced through the mini-thoracotomy access; the heart was arrested with antegrade cardioplegia. Histidine–Tryptophan–Ketoglutarate cardioplegia was used until December 2021, Del Nido cardioplegia was given in all the cases thereafter [13]. The left atrium was opened using a left atrial atriotomy in the inter-atrial groove. A right lateral approach provided a better face view of the mitral valve apparatus than median sternotomy, using atrial retractor without aid of video assistance tool. Carbon dioxide field insufflation and TOE-guided de-airing techniques were used to minimize risk of air embolus. Video supports are available at https://www.minicardiacsurgery-univpm-research.com/video-gallery/ (accessed on the 22 December 2023).

#### 2.5.2. Full Sternotomy Access

Full sternotomy access included a complete median sternotomy, opening of the pericardium and placement of usual stay sutures. Cardiopulmonary bypass was generally instituted via central arterial and bicaval cannulation. After aortic cross-clamping, antegrade histidine–tryptophan–ketoglutarate or del Nido cardioplegia was delivered in the ascending aorta. The mitral valve was exposed after a direct left atriotomy through the interatrial groove or using a trans-septal access depending on the surgeon’s preference.

### 2.6. Statistical Analysis

The patients were divided in two groups according to the surgical approach—trans-axillary and full sternotomy access. To minimize the effects of selection bias, a propensity matching was performed. The following variables were included as covariates: age (years), gender, hypertension, smoking history, history of coronary artery disease, chronic kidney disease (eGFR < 50 mL/min/1.73 m^2^), NYHA class ≥ 3, history of atrial fibrillation, left ventricular ejection fraction, pulmonary hypertension (pulmonary artery pressure ≥ 30 mmHg), tricuspid regurgitation more than moderate.

The nearest matching algorithm was applied with a calliper width for the logit of the propensity score less than 0.2. The adequacy of propensity score matching was evaluated on standardised mean difference values for each variable and was considered acceptable when the absolute value was less than 0.1. Continuous variables are expressed as means ± SDs or medians and interquartile ranges (IQRs), while categorical variables are presented as numbers and percentages. Student t or Mann–Whitney U tests and a chi-square test were used to compare continuous or categorial variables, respectively. *p*-values < 0.05 were considered statistically significant.

Survival analyses were performed with the use of Kaplan–Meier estimates and comparison between groups were made by log-rank test.

Occurrence of re-intervention on mitral valve was studied using Cumulative incidence function with death as a competing risk and Gray’s test was used for comparison between the trans-axillary and full sternotomy approach. The analysis was generated using Statistical Analysis Software (SAS), Version 3.8, SAS University Edition (SAS Institute Inc., Cary, NC, USA) and Statistical Package for Social Sciences version 27.0 (IBM SPSS Inc., Chicago, IL, USA).

## 3. Results

### 3.1. Baseline Characteristics

A total of 528 consecutive patients represented the study population, out of which 308 underwent mitral valve repair through trans-axillary approach, and 220 patients received traditional full sternotomy. Table 1 provides details about the preoperative characteristics of the unmatched populations, of the trans-axillary and of the full sternotomy cohorts. The two populations presented significant differences with patients operated on through full sternotomy access being older, more symptomatic, with a higher prevalence of chronic kidney dysfunction, pulmonary hypertension, and history of atrial fibrillation.

**Figure 1:**
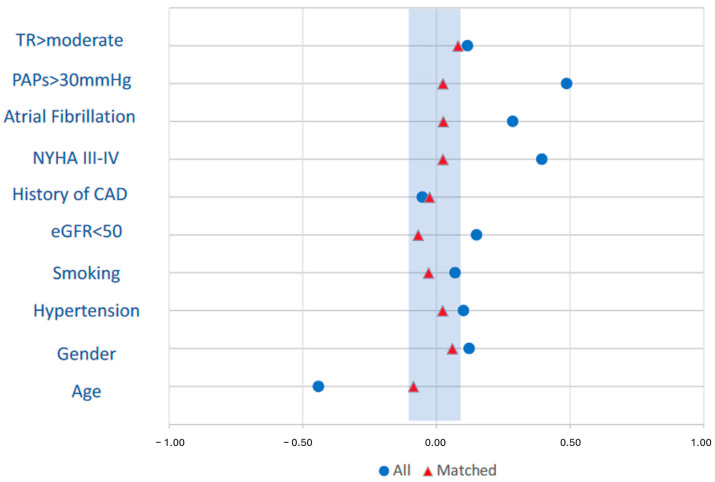
Standardised mean differences of preoperative characteristics included in the propensity match analysis before (blue) and after matching (red).

After PS match analysis, 171 pairs treated with TAxA and full sternotomy procedures were identified: the two matched cohorts appeared well balanced in terms of patients’ baseline characteristics (Figure 1, Table 1).

### 3.2. Operative Data in TAxA and FS Matched Cohorts

Resection and edge-to-edge repair were the surgical techniques more frequently used in FS access (28.1% vs. 9.4% in TAxA group, *p* < 0.001, and 25.1% vs. 11.7% in TAxA group, *p* < 0.001, respectively) while implantation of artificial chords was more commonly performed in TAxA approach (71.9% vs. 32.2% in FS group, *p* < 0.001). In the FS group, cardiopulmonary bypass time was shorter than minimally invasive group with a median of 80 min vs. 103 min *p* < 0.001; no significant difference between the two matched cohorts was found in the length of cross-clamp time (median of 64 min in FS group vs. 62 min in TAxA group, *p* = 0.5). Table 2 provides details about operative data.

### 3.3. Postoperative Outcomes in TAxA and FS Matched Cohorts

The overall 30-day mortality rate was 0.6% in both patient cohorts. There was no difference in postoperative stroke (0 vs. 0.6%; *p* = 1), respiratory failure (1.2% vs. 1.8%; *p* = 1), re-exploration for bleeding (3.5% vs. 2.4%; *p* = 0.6) and postoperative kidney disfunction (1.2% vs. 1.8%; *p* = 1) between the FS and TAxA approach, respectively. Table 3 provides details about postoperative complications.

TAxA cohort showed earlier postoperative extubation (median mechanical ventilation time was 1 h vs. 6 h in FS cohort, *p* < 0.001) with a higher rate of extubation performed in theatre soon after the end of surgical procedure (48.5% vs. 8.2%, *p* < 0.001). Compared to the FS approach, the TAxA group experienced shorter ICU stay (*p* < 0.001). There was no difference in the overall postoperative hospital stay (*p* = 0.7), but 51% of patients who had TAxA surgery were able to be discharged home without the need for any further period of cardiopulmonary rehabilitation or Cardiology work up (vs. 13.5% of patients in FS, cohort *p* < 0.001).

### 3.4. Mid-Term Outcomes in TAxA and FS Matched Cohort

**Figure 2:**
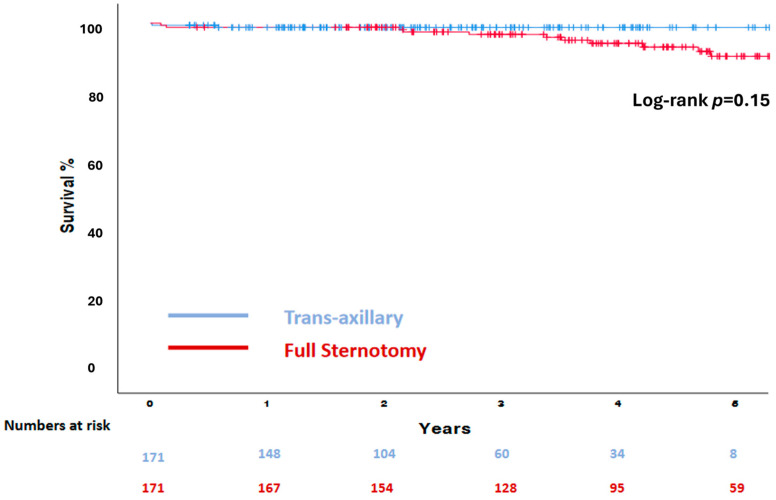
Kaplan–Meier survival analysis of the matched cohort.

Follow-up data were 100% complete at a median time of 3.4 years (2–4.8). The survival probability at 5 years was 98.8% in the TAxA cohort and 93.6% in the FS cohort (Log rank *p* = 0.15) (Figure 2).

The cumulative incidence—with death as a competing risk—of reoperation for mitral valve regurgitation was 2.6 ± 1.9% (95% confidence interval 0.43–8.7) and 4.4 ± 1.9 (95% confidence interval 1.7–9.1) at 5 years, (Gray test *p* = 0.49), respectively, in the TAxA and FS cohorts. A reoperation for mitral valve dysfunction was required in six patients in the FS cohort and in two patients in the TAxA cohort. In all patients, MV replacement was performed. Details about the mechanism of failure are reported in Table 4.

## 4. Discussion

Minimally invasive mitral valve surgery has evolved over the last two decades as an appealing alternative approach to full sternotomy due to increasing patients’ desire of less trauma and pain, quick recovery and better cosmesis.

Despite this promising and well-established evidence, minimally invasive mitral valve surgery has been slowly adopted by surgeons [4,5,14,15] due to the perception of potential higher risk of early complications secondary to prolonged operative times and difficult control of the surgical field through the small mini-thoracotomy access. Additional factors include the significant learning curve required for the surgical team to not compromise the technical results and the durability of valve repair.

There are several reasons we favour TAxA access over other well-established mini-thoracotomy approaches. TAxA provides direct vision of the mitral valve apparatus, enhancing a favourable surgical setting without increasing the complexity of the procedure and thus reducing operative times as seen in our reported values of cross-clamp and cardiopulmonary times which are significantly lower than those usually reported in mini-thoracotomy surgery experiences [1,16,17]. In addition, through a TAxA incision, the aortic valve and tricuspid valve are both accessible for repair and replacement, as is the left atrial appendage through the transverse sinus. This is well highlighted by our experience reporting that concomitant tricuspid repair and atrial fibrillation procedures were performed with similar frequency between FS and TAxA cohorts, furthermore in line as previously reported in the literature [4,18,19,20].

Trans-axillary access has been associated with outstanding early results which have largely confirmed the safety of this approach [16,21,22]. As previously reported [7], we found that both FS and TAxA approaches for mitral valve repair were characterized by a low rate of 30-day mortality (0.6% vs. 0.6%), post operative cerebral stroke (0% vs. 0.6%) and major complications. Furthermore, the TAxA cohort experienced shorter mechanical ventilation time (48.5% of patients extubated in operating theatre at the end of procedure), shorter ICU stay and reduced hospitalization with a higher percentage of patients able to be discharged home without the need for further respiratory and cardiology rehabilitation.

Since the end of 2016, we have started an integrated protocol looking at several ameliorations of different aspects of the surgical process from the preoperative to the postoperative phases. We developed an ultra-fast track protocol mainly characterized by the reduction of tissue trauma, the use of normothermic CPB, early extubation including on table extubation soon after the end of the procedures, early physiotherapy starting on Day 0 [21]. The TAxA approach has facilitated the embedding and the spreading of our ERAS evidence-based protocol by promoting quicker extubation and mobilization of the patients and shorter hospitalization stays.

The recent implementation of enhanced recovery after cardiac surgery protocols has undoubtedly the merit of focusing on the increasing demand from patients for a better surgical experience [16,17,18,23,24,25,26]. Nevertheless, guaranteeing long-term durable results remains one the essential peculiarities of surgical mitral valve repair. Data from medium- and long-term longitudinal evaluation of patients who underwent mitral valve repair trough minimally invasive approach are still lacking. We were able to present our mid-term results with 100% complete follow-up. Overall survival was excellent; 5-year survival was 98.8% in the TAxA cohort and 93.6% in the FS cohort, similar to previous reports [4,27,28]. This finding is not surprising since our population included patients who were young/middle-aged with few comorbidities, suffering generally from degenerative mitral valve disease and operated on an earlier stage of heart failure [29]. In our cohorts, the need for mitral valve reoperation was very low: the cumulative incidence of reoperation was 2.6% and 4.4% at 5 years, respectively, in TAxA and FS cohorts, considering death as a competing risk. Within the limitations of considering different surgical populations and the inclusion of different lesions, we found our durability results comparable with other series of mitral valve repair [30,31,32,33]. Over the years, mitral valve repair has become the gold-standard treatment of mitral valve regurgitation in expert high-volume centres, especially in degenerative disease, as highlighted by David et al. in a large series at 20 years follow-up [32]. The late outcomes reported in the literature, referred mostly to the traditional FS approach and further investigations are needed to confirm the durability of MV repair trough trans-axillary access.

This study presents some limitations. It is a single-centre retrospective analysis on prospectively collecting data and the generalization of results are confined to our institutional experience. There were several differences in baseline characteristics between TAxA and FS populations; however, the propensity match analysis returned two well-balanced cohorts of patients. The reoperation events included patients referred to our centre and did not exclude a possible underestimation because of missed data (patients who received reintervention in another cardiac surgery department). Furthermore, the hard endpoint of reoperation is not the ideal marker to weight the failure of valve repair over time; reoperation is a clinical decision, and the unavailability of serial echocardiography evaluation represents a further limitation of our follow-up.

## 5. Conclusions

The TAxA approach for mitral valve repair was associated with very low in-hospital mortality and low rate of major postoperative complications with values that are at least similar to those derived from a matched cohort of patients undergoing FS surgery. Patients operated on through minimally invasive access experienced earlier extubation, shorter ICU stay and reduced hospitalization with a higher rate of patients able to be discharged straight home with no need for further rehabilitation. In the mid-term, TAxA was furthermore associated with excellent survival and low rate of mitral valve reoperation.

## Figures and Tables

**Table 1:** Preoperative patients’ characteristics in unmatched and matched populations.

		Unmatched			Matched		
Variables	Overall *n* = 528	FS *n* = 220	TAxA *n* = 308	Absolute SMD	FS *n* = 171	TAxA *n* = 171	Absolute SMD
	Mean ± SD *n* (%)	Mean ± SD *n* (%)	Mean ± SD *n* (%)		Mean ± SD *n* (%)	Mean ± SD *n* (%)	
Age (years)	64 ± 11	67.2 ± 11	62 ± 11	0.44	66 ± 10	65 ± 11	0.09
Gender (M/F)	194/334	88/132	106/202	0.13	105/66	110/61	0.06
BMI (kg/m^2^)	25.1 ± 4.1	25.5 ± 4.2	24.9 ± 3.9	0.15	25.4 ± 4.2	24.9 ± 4.3	0.12
BSA (m^2^)	1.85 ± 0.22	1.84 ± 0.21	1.86 ± 0.22	0.09	1.83 ± 0.21	1.84 ± 0.22	0.05
Hypertension	288 (54.5)	127 (57.7)	161 (52.3)	0.10	97 (56.7)	95 (55.6)	0.02
Diabetes Mellitus	35 (6.6)	14 (6.4)	21 (6.8)	0.02	11 (6.4)	11 (6.4)	0
Dyslipidemia	161 (30.5)	77 (35)	84 (27.3)	0.16	55 (32.2)	51 (29.8)	0.05
Smoking history	99 (18.8)	45 (20.5)	54 (17.5)	0.07	34 (19.9)	36 (21.1)	0.03
CKD (eGFR < 50 mL/min/1.73 m^2^)	68 (12.9)	35 (15.9)	33 (10.8)	0.15	22 (12.9)	26 (15.2)	0.07
Previous cerebral stroke	9 (1.7)	4 (1.8)	5 (1.6)	0.02	2 (1.2)	2 (1.2)	0
Previous CAD	30 (5.7)	11 (5)	19 (6.2)	0.05	9 (5.3)	10 (5.8)	0.03
Previous PCI	18 (3.4)	9 (4.1)	9 (2.9)	0.07	6 (3.5)	5 (2.9)	0.03
NYHA class ≥ III	207 (39.2)	111 (50.5)	96 (31.2)	0.39	76 (44.4)	74 (43.3)	0.02
Permanent pacemaker	11(2.1)	7 (3.2)	4 (1.3)	0.13	3 (1.8)	3 (1.8)	0
History of AF	148 (28)	78 (35.5)	70 (22.7)	0.29	51 (29.8)	49 (28.7)	0.03
Preoperative AF	148 (28)	79 (36)	69 (22.4)	0.3	50 (29.2)	49 (28.7)	0.01
Hemoglobin (g/dL)	13.6 ± 1.5	13.5 ± 1.6	13.7 ± 1.5	0.13	13.6 ± 1.5	13.5 ± 1.5	0.07
Hematocrit (%)	41.1 ± 4.4	40.9 ± 4.5	41.3 ± 4.4	0.09	41.3 ± 4.3	41 ± 4.2	0.07
Preoperative ventricular arrhythmia	10 (1.9)	2 (0.9)	8 (2.6)	0.13	2 (1.2)	4 (2.3)	0.08
LVEF (%)	61.2 ± 7.9	60.9 ± 9.3	61.4 ± 6.7	0.06	61.2 ± 9	61 ± 8	0.02
PAPs ≥ 30 mmHg	230 (43.6)	126 (57.3)	104 (33.9)	0.48	85 (49.7)	83 (48.5)	0.02
TVR ≥ moderate	129 (24.4)	60 (27.3)	69 (22.4)	0.11	48 (28.1)	42 (24.6)	0.08
EuroSCORE II (%)	1.24 ± 1.1	1.4 ± 1.1	1.13 ± 0.9	0.12	1.29 ± 1.2	1.23 ± 0.9	0.08

AF, atrial fibrillation; BMI, body mass index; BSA, body surface area; CAD, coronary artery disease; CKD, chronic kidney disease; eGFR, glomerular filtration rate; LVEF, left ventricular ejection fraction; NYHA, New York Heart Association; PAPs, systolic pulmonary artery pressure; PCI, percutaneous coronary intervention; SMD, standardised mean difference; TVR, tricuspid valve regurgitation.

**Table 2:** Operative data.

Variables	Matched		
	FS *n* = 171	TAxA *n* = 171	*p*
	Median [IQR] *n* (%)	Median [IQR] *n* (%)	
Mitral repair for PMVL prolapse	82 (48)	103 (60.2)	0.03
Mitral repair for AMVL prolapse	19 (11.1)	10 (5.8)	0.1
Mitral repair for bileaflets disease	35 (20.5)	35 (20.5)	1.0
Neochordae	55 (32.2)	123 (71.9)	<0.001
Leaflet(s) resection	48 (28.1)	16 (9.4)	<0.001
Edge-to-edge	43 (25.1)	20 (11.7)	<0.001
Concomitant TV repair	33 (19.3)	20 (11.7)	0.08
Concomitant AF surgery	2 (1.2)	4 (2.3)	0.7
Cardiopulmonary bypass time (minutes)	80 (69–94)	103 (87–123)	<0.001
Cross-clamp time (minutes)	64 (55–75)	62 (51–78)	0.5
Repeated Cross-clamp	4 (2.3)	5 (2.9)	1.0
Intraoperative SAM	2 (1.2)	4 (2.3)	0.7
Intraoperative LCx occlusion	0	1 (0.6)	1.0
Intraoperative aortic dissection	0	1 (0.6)	1.0

AF, atrial fibrillation; AMVL, anterior mitral valve leaflet; LCx, left circumflex artery; PMVL, posterior mitral valve leaflet; SAM, systolic anterior motion; TV, tricuspid valve.

**Table 3:** Postoperative data.

Variables	Matched		
	FS *n* = 171	TAxA *n* = 171	*p*
	Median (IQR) *n* (%)	Median (IQR) *n* (%)	
30-day mortality	1 (0.6)	1 (0.6)	1.0
Cerebral stroke	0 (0)	1 (0.6)	1.0
Post-operative myocardial infarction	3 (1.8)	3 (1.8)	1.0
CVVHD	1 (0.6)	1 (0.6)	1.0
Bleeding re-thoracotomy	6 (3.5)	4 (2.4)	0.6
On table extubation	14 (8.2)	83 (48.5)	<0.001
Mechanical ventilation time (hours)	6 (4–10) 12 ± 39	1 (0–6) 5 ± 11	<0.001
Respiratory failure	2 (1.2)	3 (1.8)	1.0
ICU stay (hours)	25 (24–48) 43 ± 40	24 (20–44) 36 ± 42	<0.001
New onset AF (preoperative SR)	35 (28.9)	31 (25.4)	0.6
Permanent pacemaker	12 (7.1)	8 (4.8)	0.5
Deep wound complication	2 (1.2)	0 (0)	0.5
Pre-discharge red blood cells transfusion (number of patients)	34 (20.1)	26 (15.8)	0.3
Hospital stay (days)	7 (6–8)	7 (6–8)	0.7
Discharge Home	23 (13.5)	86 (50.6)	<0.001
LVEF (%)	55 (50–60)	55 (50–60)	0.6
Residual moderate MR	1 (0.6)	5 (2.9)	0.1
Redo for early failure	5 (2.9)	2 (1.2)	0.4

AF, atrial fibrillation; CVVHD, continuous veno-venous haemodialysis; ICU, Intensive Care Unit; LVEF, left ventricular ejection fraction; MR, mitral regurgitation; SR, sinus rhythm.

**Table 4:** Aetiology of mitral valve reoperation after mitral valve repair.

Patient	Previous MV Surgical Approach	Type of Repair Technique	Mode of Failure	Interval From Repair, y
1	FS	AMVL neochordae implantation	New onset of AMVL prolapse	1.94
2	FS	AMVL neochordae implantation	Recurrent AMVL prolapse	2.49
3	FS	PMVL neochordae implantation	Endocarditis: perforation and new onset of PMVL prolapse	0.48
4	FS	PMVL resection	New onset of flail leaflet due to chordal rapture	3.96
5	FS	Edge-to-edge	Recurrent AMVL and PMVL prolapse	0.49
6	FS	Edge-to-edge	New onset of AMVL prolapse	4.49
7	TAxA	PMVL neochordae implantation	Recurrent PMVL prolapse and chordal rapture	3.36
8	TAxA	PMVL neochordae implantation	New onset of PMVL chordal rapture	1.86

AMVL, anterior mitral valve leaflet; PMVL, posterior mitral valve leaflet; FS, full sternotomy; TAxA, transaxillary access.

## Data Availability

The data underlying this article will be shared on reasonable request to the corresponding author.
